# Diagnosing Rhodococcus equi Infections in a Setting Where Tuberculosis Is Highly Endemic: a Double Challenge

**DOI:** 10.1128/JCM.02284-14

**Published:** 2015-03-18

**Authors:** Thuy Le, Shama Cash-Goldwasser, Phan Vinh Tho, Nguyen Phu Huong Lan, James I. Campbell, H. Rogier van Doorn, Nguyen Tien Lam, Nguyen Vu Trung, Dao Tuyet Trinh, Nguyen Van Kinh, Heiman F. L. Wertheim

**Affiliations:** a Oxford University Clinical Research Unit, Wellcome Trust Major Overseas Program, Ho Chi Minh City, Vietnam; b Centre for Tropical Medicine, Nuffield Department of Clinical Medicine, University of Oxford, Oxford, United Kingdom; c McGill University Faculty of Medicine, Montreal, Quebec, Canada; d Hospital for Tropical Diseases, Ho Chi Minh City, Vietnam; e National Hospital for Tropical Diseases, Hanoi, Vietnam; f Hawaii Center for AIDS, University of Hawaii at Manoa, Honolulu, Hawaii, USA

## Abstract

Rhodococcus equi infection is increasing in regions with high HIV prevalence worldwide. The microbiological features and clinical mimicry of tuberculosis infection pose diagnostic challenges in high-tuberculosis-incidence settings. We present two HIV-associated cases of R. equi infection from Vietnam and discuss the unique diagnostic challenges in such settings.

## CASE REPORTS

### Case 1.

**FIG 1:**
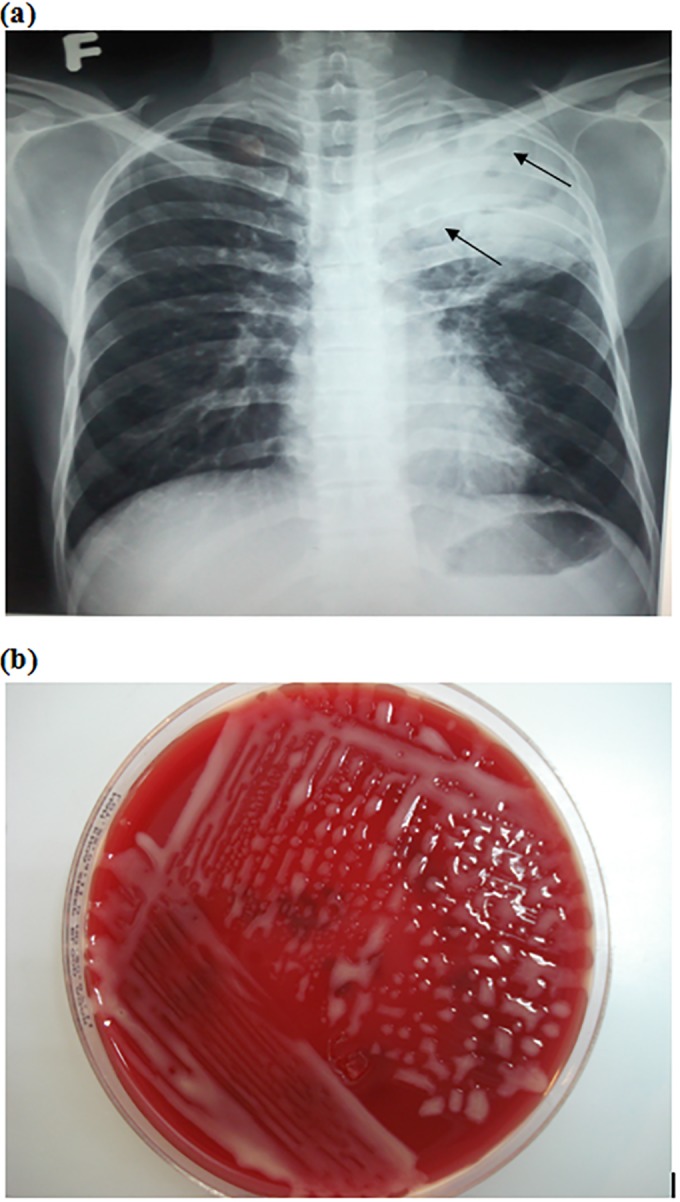
(a) Chest X-ray of patient 1 with arrows indicating several cavitary lesions within the left upper lobe consolidation; (b) salmon-pink colonies after 7-day incubation of the pleural fluid, plated on Colombia blood agar.

A 27-year-old male was referred to the National Hospital for Tropical Diseases in Hanoi, Vietnam, in 2011 with 2 months of fever, productive cough, and trouble breathing. He was diagnosed with HIV in 2006 but had not started antiretroviral therapy (ART). He worked as a nurse and had not had contact with a farming environment or animals. The physical examination revealed a temperature of 39°C, labored breathing, and lung crackles. Laboratory testing showed normal electrolytes, transaminases, and creatinine but low hemoglobin (9.4 g/dl) (reference range, 14.5 to 15.7 g/dl) and elevated lactase dehydrogenase (452 IU/liter) (reference range, 140 to 280 IU/liter). The CD4 count was 36 cells/μl (reference range, 500 to 1,200 cells/μl). Sputum stains revealed Gram-positive bacilli and were negative for acid-fast bacilli (AFB) and fungi. A chest X-ray showed a left upper lobe consolidation with cavitary lesions (Fig. 1a) and a small pleural effusion. A thoracentesis was performed, and the pleural fluid showed inflammatory exudates and Gram-positive coccobacilli but no AFB or fungi. Blood culture on the day of admission showed no growth. The patient did not improve after 1 week of intravenous piperacillin-tazobactam at 3.375 g every 6 h and oral metronidazole at 500 mg three times daily. On hospitalization day 10, the smooth mucoid salmon-pink appearance of the pleural fluid culture raised suspicion for Rhodococcus equi (Fig. 1b). The organism was identified by using API identification strips (bioMérieux, Marcy l'Etoile, France) and was confirmed by 16S rRNA sequencing. Subsequent blood culture (hospitalization day 21) also grew R. equi. Susceptibility testing was performed by disk diffusion method using Oxoid (Oxoid, Cheshire, United Kingdom) pills on sheep blood agar (Merck) using CLSI cutoffs for Staphylococcus aureus. The isolate was susceptible to vancomycin, ampicillin-clavulanate, rifampin, cefoxitin, cotrimoxazole, erythromycin, gentamicin, levofloxacin, and ciprofloxacin and was resistant to oxacillin, clindamycin, penicillin, and ampicillin. The patient was started on oral rifampin at 450 mg daily, levofloxacin at 750 mg daily, and erythromycin at 500 mg twice daily based on susceptibility testing. His fever and respiratory symptoms gradually improved over 4 weeks of inpatient treatment. He was started on ART and was discharged home after 6 weeks of hospitalization; the patient was subsequently lost to follow-up.

### Case 2.

**FIG 2:**
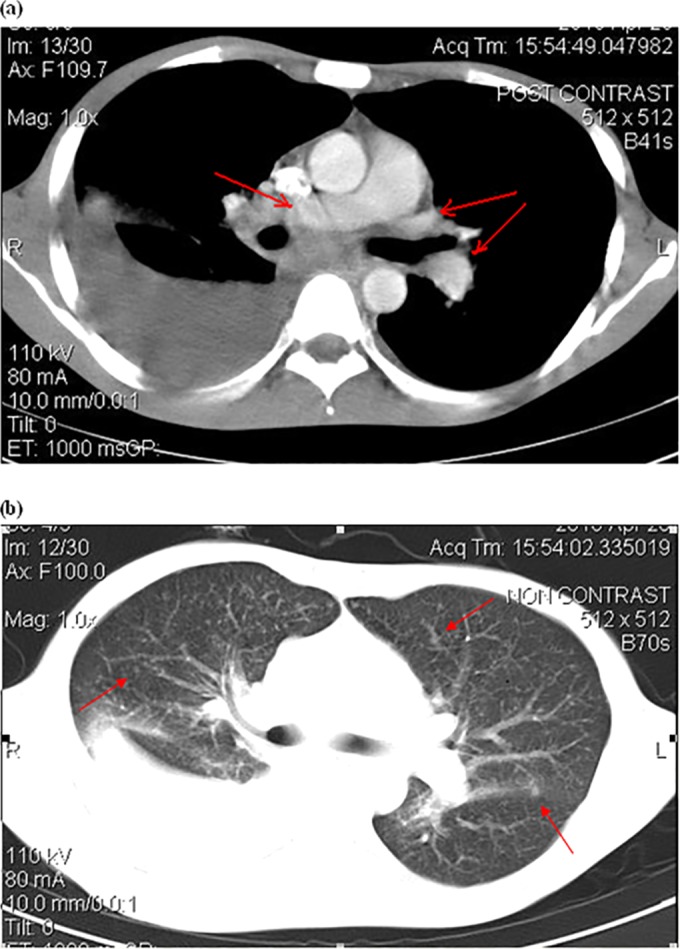
Chest computed tomography of patient 2 showing a large pleural effusion in the right lung. (a) Arrows indicate enlarged mediastinal lymph nodes; (b) arrows indicate parenchymal nodules.

A 28-year-old male was admitted to the Hospital for Tropical Diseases in Ho Chi Minh City, Vietnam, in 2012 with 2 months of fever, weight loss, and nonproductive cough. The patient had been working for 5 years in a greenhouse with frequent manure exposure. The physical examination showed a temperature of 38°C, oral thrush, and enlarged cervical lymph nodes of 2 to 3 cm. Laboratory testing showed electrolytes, transaminases, and creatinine levels within reference ranges but low hemoglobin (7.3 g/dl). The results of three different enzyme-linked immunosorbent assays (ELISAs) for HIV were positive, and the CD4 count was 61 cells/μl. Multiple sputum stains for bacteria and AFB were negative. A chest X-ray showed interstitial changes with a small right pleural effusion. The patient was treated with intravenous ceftriaxone at 2 g daily and oral cotrimoxazole at 160 mg (trimethoprim)/800 mg (sulfamethoxazole) four times daily for presumed bacteria and Pneumocystis jirovecii pneumonia for 1 week without clinical improvement. Blood culture on the day of admission showed no growth. A chest computed tomography showed mediastinal lymphadenopathy, numerous parenchymal nodules, and a right pleural effusion (Fig. 2). A thoracentesis was performed, and the pleural fluid showed inflammatory exudates but was negative for Gram and Ziehl-Neelsen stains. Tuberculosis was the leading diagnosis, and the patient was empirically started on antituberculosis therapy with isoniazid at 300 mg daily, rifampin at 450 mg daily, pyrazinamide at 1 g daily, and ethambutol at 800 mg daily. Pleural fluid culture on hospitalization day 7 grew mucoid colonies of weakly acid-fast coccobacilli. The colonies were identified as R. equi by using API identification strips. Despite the identification of R. equi, the treating clinicians refused to stop antituberculosis therapy. Upon discussions with infectious disease specialists, levofloxacin at 750 mg daily was added to provide dual coverage for tuberculosis and R. equi. Fever and cough resolved after 1 week. The patient completed a 6-month course of therapy and remained well at month 18 of follow-up. His CD4 count rose to 465 cells/μl on ART.

R. equi is increasingly recognized as a human pathogen, with approximately 200 cases described in the literature (1). Eighty-five percent of R. equi infections in humans occur in immunocompromised patients, with patients with HIV accounting for two-thirds of cases (2). Approximately 50% of patients have some contact with herbivores or their manure (3, 4). Mortality is 50% in HIV-infected patients and is more than twice the mortality in HIV-uninfected patients (3, 5). Most patients present with subacute onset of pulmonary disease, chest radiographic abnormality, and constitutional symptoms (1, 3). The majority of HIV-infected patients have concurrent bacteremia and may develop disease at multiple distant sites (1, 5). Chest radiographs are abnormal in 95% of patients, and the most common radiographic findings are cavitary lung lesions (75%) and pleural effusion (20%) (4, 11). Diagnosis can be made via culture of biopsy specimens and all bodily fluids (1).

On the basis of morphology, several microbiological features of R. equi allow it to escape identification in a microbiology laboratory. First, R. equi grows readily on nonselective medium; however its variable rod-to-coccus appearance can be mislabeled as “mixed flora” on Gram-stained sputum. Early colonies have a pleomorphic diphtheroid appearance, which can easily be dismissed as a contaminant. Further, the organism can be acid fast on a Ziehl-Neelsen smear and be mistaken for a mycobacterium (1, 3, 5). R. equi was found to have the highest sequence homology with Mycobacterium tuberculosis in a comparison with other bacterial genera or species based on partial genome sequencing (6). The close genetic distance may explain similarities in disease pathogenesis, in particular the ability of R. equi to persist and multiply inside macrophages (7) and to cause pyogranulomatous pneumonia in humans. On clinical grounds, human R. equi infection is indistinguishable from tuberculosis and hence may be misdiagnosed in settings of high tuberculosis prevalence. Vietnam is among those countries with the highest tuberculosis burden in the world (8), with tuberculosis being the most common HIV-associated diagnosis (9). Our patients presented with clinical features characteristic of tuberculosis, and one patient was started on antituberculosis treatment based on clinical suspicion. First-line tuberculosis therapy exposes R. equi-infected patients to rifampin monotherapy, which promotes development of resistance and treatment failure (10). R. equi is inherently resistant to multiple antibiotics (3, 12, 13), and long-term therapy with two to three drugs is recommended particularly for immunocompromised patients (1). The duration of therapy depends upon the extent of infection, clinical and radiographical resolution, and immune status (1). Most experts recommend at least 6 months of therapy for immunocompromised patients (1). Adding a fluoroquinolone to the standard four-drug antituberculosis regimen provided antimicrobial coverage for both R. equi and M. tuberculosis, which was more acceptable to the clinicians caring for the patient, and in this case resulted in good therapeutic outcome. In both patients, immune reconstitution on ART was likely important to disease resolution.

Our case report highlights the challenge in diagnosing R. equi infections in resource-limited settings with high tuberculosis and HIV prevalence. Increased awareness of the disease and its diagnostic challenges may prevent misdiagnoses and may improve the management of R. equi infections in these settings.
